# Promise and pitfalls in the application of big data to occupational and environmental health

**DOI:** 10.1186/s12889-017-4286-8

**Published:** 2017-05-09

**Authors:** David M. Stieb, Cécile R. Boot, Michelle C. Turner

**Affiliations:** 1 Population Studies Division, Environmental Health Science and Research Bureau, Health Canada, 420-757 West Hastings St. - Federal Tower, Vancouver, BC V6C 1A1 Canada; 2 School of Epidemiology, Public Health and Preventive Medicine, University of Ottawa, Ottawa, Canada; 3 Department of Public and Occupational Health, Amsterdam Public Health Research Institute, VU University Medical Center Amsterdam, Van der Boechorststraat 7, 1081 BT Amsterdam, The Netherlands; 4 Barcelona Institute for Global Health (ISGlobal), 4c/ Rosselló, 132, 5th 2nd, 08036 Barcelona, Spain; 5 Universitat Pompeu Fabra (UPF), Barcelona, Spain; 6 CIBER Epidemiología y Salud Pública (CIBERESP), Madrid, Spain; 7 McLaughlin Centre for Population Health Risk Assessment, University of Ottawa, Ottawa, ON Canada

Is “big data” merely a catchphrase, or does the approach hold real promise in informing occupational and environmental health? Can challenges related to messy and unrepresentative data and spurious findings be overcome?

## Promise

The potential power of big data to inform public health decision-making has been widely recognized [1, 2]. However, there is a paucity of published primary research employing these methods in this journal and elsewhere [3, 4]. The *American Journal of Public Health* encouraged new research in this area and recently appointed an inaugural associate editor for digital health [3].

Big data are typically defined in relation to the “three Vs”, volume, velocity and variety (and more recently, variability, veracity and value) [5]. Other defining characteristics include the emergence of new data sources and providers such as social media, mobile applications and wearable technology such as fitness trackers (the “quantified self” [6]), the need for new analytical methods such as machine learning, non-traditional multi-disciplinary partnerships and real-time analysis and forecasting [7].

Along similar lines, sharing of clinical trial and other study data has also been advocated as a means of broadening access to and more fully exploiting the collective power of data. In addition to increasing statistical power, which could potentially facilitate detecting small signals earlier, which may be particularly important in environmental health, advantages of pooling data include enhanced ability to examine heterogeneity between diverse populations, and consideration of novel hypotheses not tested by the original investigators [8]. Data sharing initiatives must overcome barriers including providing protections for original investigators, particularly those in low-resource countries [9], and issues related to data ownership, privacy and security [8]. The Healthy Birth, Growth, and Development–Knowledge Integration initiative is an example of a data sharing initiative which has navigated many of these issues [8]. A need has also been identified to address barriers to the international sharing of routinely collected public health data, including technical, motivational, economic, political, legal and ethical factors [10].

Exposure analysis is the keystone of occupational and environmental health. As a result, the concept of big data in this context is linked closely to that of the exposome, the totality of human environmental, occupational and other exposures from conception to death [11]. These exposures interact with other determinants of internal dose and health effects characterized by their own data-rich “omes” – the genome, metabolome, lipidome, transcriptome and proteome, among others, analysis of all of which requires novel data analysis methods [11–14]. The exposome may be characterized using a vast array of methods including measurement of both exogenous and endogenous biomarkers in biological specimens, direct environmental monitoring using dedicated sensors, and indirect sources such as operational data from metering and energy use, and facilities management data [12, 15–17].

## Pitfalls

As a counterpoint to the potential of big data, one of the primary concerns is the potential for spurious findings, (described at their worst as “fanciful rubbish” or “big error”) that can be generated by employing “much bigger and messier data” [2, 7]. Related to these limitations of big data are epistemological issues around the approach to how they are analyzed and how knowledge is generated. Some have gone so far as to argue that big data analytics allow the data to “speak for themselves,” free of a priori hypotheses, and by extension of investigator bias, but others have countered that whether desirable or not, this is unattainable since all data are in fact framed by the methods and constructs under which they are collected [2, 18]. A hybrid approach has been advanced where big data analysis, machine learning or “knowledge discovery” is guided by theory and practical experience, including a more selective approach to choosing appropriate data sources and analysis methods, as well as ultimately testing hypotheses generated from initial analyses [2, 18]. An additional concern is that to the extent that big data relies on consumer “data trails,” mobile devices, wearable technology or electronic medical records, they may exclude those with limited footprints owing to barriers related to age, race, socioeconomic status, access to care or health literacy [5]. This has the potential to amplify environmental injustice concerns to the extent that it further disadvantages populations who already experience a disproportionate health burden related to environmental exposures [19].

## Application to occupational and environmental health

Notwithstanding these important caveats, the potential for big data to inform public health and occupational and environmental health more specifically has been recognized by several funding agencies. The National Institute of Environmental Health Sciences is part of a National Institutes of Health-wide data science initiative, “Big data to knowledge” (BD2K), which aims to facilitate wide use of data, develop methods, software and tools, build capacity through training, and support data infrastructure [20]. The European Commission recently issued a call for proposals pertaining to “Big data supporting Public Health Policies,” focusing on “how to better acquire, manage, share, model, process and exploit” big data for public health purposes, highlighting the opportunities they may provide to identify interactions between environmental, genetic and behavioral determinants of health [21]. Funded initiatives include the European Exposome Cluster [22], US Health and Exposome Research Center: Understanding Lifetime Exposures (HERCULES) [23], and the CANadian Urban Environmental (CANUE) Health Research Consortium [24].

Research in both occupational and environmental health has made widespread use of large datasets for many years. It is instructive to consider how it has been transformed by increasing application of big data and data sharing. In the environmental health realm, there is a long history in air pollution epidemiology of combining routinely available administrative health or vital statistics data, with environmental monitoring data, particularly to examine effects of short term variability in exposure using time-series or case-crossover analysis [25]. This approach was subsequently applied to examining the effects of long term exposure by linking an existing cohort, the American Cancer Society cohort [26], to routinely available environmental data, in order to relatively inexpensively replicate findings from a dedicated cohort study, the Six Cities Study [27]. This approach has now been applied to many other cohorts, and further by creating synthetic cohorts by linking census or tax data to vital statistics data and incorporating spatially comprehensive exposure data combining ground based monitoring, satellite observations, chemical/meteorological models and land use patterns [28, 29]. There are also examples of exploiting clinical trial data to examine associations with air pollution, unrelated to the original study hypothesis, e.g. linking clinical data on carotid intima media thickness as a measure of development of atherosclerosis, to air pollution exposure [30]. While social media as a source of big data have been dismissed as “frivolous,” in addition to being used to track communicable disease for surveillance purposes, there are examples of application to chronic disease and environmental health such as development of predictive models of asthma using Twitter, Google searches and air monitoring data [31]. Asthma exacerbations are well documented in relation to air pollution exposure, and asthma also lends itself to “self-quantification” in relation to tracking of lung function and symptoms. Licksai et al. [32] developed a mobile application which combines these features of asthma with air quality forecasts and advice.

Similarly, in occupational health, workplace injury and illness data from physician reporting, employer records and workers compensation claims have been a longstanding resource for research and surveillance. Recently, the US Occupational Safety and Health Administration strengthened reporting requirements and improved public access to these data, motivated partly by increasing the utility of the data for research [33]. In Europe, investigators employed 20 physician reporting and compensation claim datasets from 10 countries to examine trends in occupational disease incidence, accounting for the diversity of data collection methods employed in each country, and demonstrated the potential of data sharing in this area [34]. A key aim of exploiting these data is to improve the capacity to predict and prevent injury and disease in the workplace [35]. Evaluating longer term sequelae of workplace disease and injury requires different types of data. Scandinavia has a long tradition of linking cohort studies to register data to gain insight into predictors of sick leave and work disability [36]. The social security system is a determining factor for the content of registers and there may be important differences between countries. While sick leave benefits are taken over by the social security system in Scandinavia relatively early in the process, in contrast in the Netherlands, the employer is responsible for payment of salary during the first two years of sick leave. As a result, there is no national registration of sick leave, which is a disincentive for employers for valid company registration, reducing its validity as a measure. Nonetheless, first attempts are being made in the Netherlands to link occupational health cohort data to national registers that are a reliable source for measures related to source of income [37]. Social security data have also been widely used to examine work disability benefits and transitions from work to retirement.

## Conclusions

Big data and data sharing have the potential to inform occupational and environmental health by exploiting innovations related to non-traditional data sources or providers and novel partnerships. Promising applications include real time analysis and forecasting, and innovative analyses of clinical trial or observational data originally collected for other purposes. However, in order to support these innovations, advances are also required in data curation, protection of privacy and security, as well as data analysis methods. Challenges related to messy and unrepresentative data and spurious findings, as well as epistemological issues and equity considerations must also be addressed.

## Abbreviations

BD2K: Big Data to Knowledge
CANUE: Canadian Urban Environmental Health Research Consortium)
HERCULES: Health and Exposome Research Center: Understanding Lifetime Exposures
